# MegaD: Deep Learning for Rapid and Accurate Disease Status Prediction of Metagenomic Samples

**DOI:** 10.3390/life12050669

**Published:** 2022-04-30

**Authors:** Yassin Mreyoud, Myoungkyu Song, Jihun Lim, Tae-Hyuk Ahn

**Affiliations:** 1 Program in Bioinformatics and Computational Biology, Saint Louis University, Saint Louis, MO 63104, USA; yassin.mreyoud@slu.edu; 2 Department of Computer Science, University of Nebraska Omaha, Omaha, NE 68182, USA; myoungkyu@unomaha.edu; 3 Saint Paul Preparatory, Seoul 06593, Korea; jihunlim24@gmail.com; 4 Department of Computer Science, Saint Louis University, Saint Louis, MO 63104, USA

**Keywords:** metagenomics, deep learning, phenotype prediction, sample classification

## Abstract

The diversity within different microbiome communities that drive biogeochemical processes influences many different phenotypes. Analyses of these communities and their diversity by countless microbiome projects have revealed an important role of metagenomics in understanding the complex relation between microbes and their environments. This relationship can be understood in the context of microbiome composition of specific known environments. These compositions can then be used as a template for predicting the status of similar environments. Machine learning has been applied as a key component to this predictive task. Several analysis tools have already been published utilizing machine learning methods for metagenomic analysis. Despite the previously proposed machine learning models, the performance of deep neural networks is still under-researched. Given the nature of metagenomic data, deep neural networks could provide a strong boost to growth in the prediction accuracy in metagenomic analysis applications. To meet this urgent demand, we present a deep learning based tool that utilizes a deep neural network implementation for phenotypic prediction of unknown metagenomic samples. (1) First, our tool takes as input taxonomic profiles from 16S or WGS sequencing data. (2) Second, given the samples, our tool builds a model based on a deep neural network by computing multi-level classification. (3) Lastly, given the model, our tool classifies an unknown sample with its unlabeled taxonomic profile. In the benchmark experiments, we deduced that an analysis method facilitating a deep neural network such as our tool can show promising results in increasing the prediction accuracy on several samples compared to other machine learning models.

## 1. Introduction

Recent research on microbial communities has actively studied the topic of metagenomics of environmental sample diversity while focusing on the role of microbes in human, animal, plant, and environmental niches [1]. Analysis of metagenomic data related to human health and disease has surged in popularity due to the increasing availability of metagenomic sequencing data, which is the result of decreasing sequencing costs. Several projects, such as the MetaHIT consortium and the Human Microbe Project, aimed to catalogue metagenomic differences in healthy and sick individuals utilizing whole genome sequencing techniques [2,3]. These large-scale metagenomics projects provide extensive publicly available datasets that allow research groups to delve much deeper into specific disease–microbe relationships. However, most of these studies rely only on statistical analysis or on simple machine learning approaches.

As the sheer volume of features in samples becomes ever larger, the two main challenges of analyzing metagenomic data are as follows. (1) First, each sample may contain thousands of species of widely varying abundances. (2) Second, the sequencing of metagenomic data is non-trivial due to the abundance of replicated sequences scattered across many different species. To identify species abundance within a sample, a recent study performed 16S rRNA gene amplicon analysis which amplifies the 16S rRNA region to identify reads hailing from different species [4]. These reads are then assigned to specific taxonomies as taxonomic profiles are created. The association between read and taxonomy is defined as an operational taxonomic unit, or OTU. The main limitation of 16S rRNA is that OTUs commonly have the phylum or genus taxonomic resolution, making species and strain level analysis difficult [5]. In recent years, the decreasing cost of whole genome sequencing (WGS) has led to increased adoption of this technique for metagenomic analysis. In species and strain level classification, WGS provides greater extendibility and flexibility than rRNA sequencing. WGS also enables the classification of viral samples, while 16S rRNA sequencing does not [6]. At present, both sequencing techniques are in use depending on the specific application at hand [7]. On the one hand, 16S rRNA sequencing is cheaper and more applicable at a large scale, while on the other WGS is suited to smaller sample sizes since it provides a much higher resolution albeit at an increased cost [8].

Another important aspect of metagenomic analysis is taxonomic classification. Taxonomic classification is the process of identifying microbial presence and abundance by comparing sequenced reads to a database of catalogued genomes. Many tools have been developed to perform this task, such as QIIME (QIIME 2), which classifies 16S rRNA samples using an OTU binning method [9]. Existing tools and techniques classify WGS sequences by (1) searching reads against reference genomes [10,11,12], (2) using short genomic substrings (*k*-mers) [13,14,15], and (3) aligning reads to specific genetic markers [16,17]. In modern metagenomics, several approaches leverage taxonomic classification to identify linkages between diseases and microbes. For example, the projects DIABIUMME and MetaSub aim at linking microbial flora to their environments [18,19,20,21]. These approaches in metagenomics are suited for machine learning algorithms since a deep analysis of patterns within samples is required for extracting patterns from large datasets [22,23].

Currently, there are several machine learning based tools to analyze these metagenomic datasets for taxonomic classification. MetAML takes advantage of several machine learning models to link taxonomic profiles to specific phenotypes [24]. MetaDprof uses spline regression to identify differential abundances of samples [25]. MegaR leverages random forest, generalized linear models, and support vector machines to try and predict host phenotype from taxonomic profiles generated from metagenomic data [26]. PopPhy utilizes a convolutional neural network and tree structure for its phenotypic prediction [27]. DeepMicro is another deep representation learning framework that transforms a high-dimensional microbiome profile to a low-dimensional representation [28]. Although these tools are useful for examining microbiome–phenotype associations, there are important limitations within these tools. MetAML cannot support 16S rRNA sequencing data. MetaDprof is effective for longitudinal studies, but does not provide proper performance for general sample classification. MegaR and PopPhy struggle to function properly on extremely large datasets. DeepMicro often drops important features by transforming high-dimensional profiles to low-dimensional representations. 

The idea of metagenomic analysis is to identify a link between the microbial diversity of a given sample and the phenotype of the host. Previous metagenomic analysis approaches primarily utilized statistical methods which were time consuming and incapable of processing large amounts of data [29]. More recent approaches have applied machine learning models, such as support vector machines, generalized linear models, and random forest, in order to identify microbiome host links [26]. Although these approaches have shown respectably accurate results on many datasets, they still revealed key limitations to be solved urgently. First, these approaches have repeatedly failed to show appropriate performance on datasets with more than two levels of classification, which presents a significant limitation in the usefulness of these models. Second, these models are unable to classify large datasets with thousands of features and samples.

To address these limitations, we propose a deep learning based approach for applying deep neural networks for this classification problem. We have developed a deep learning based tool, MegaD ( (accessed on 27 April 2022)) which examines the links between microbes and their hosts and performs precise phenotypic classification on metagenomic samples. The main contributions made by MegaD are as follows:

1. MegaD is developed based on deep neural networks for phenotypic prediction of unknown metagenomic samples, supporting both 16S rRNA and WGS data;
2. MegaD demonstrates the robustness of handling extremely large datasets containing thousands of features and samples;
3. MegaD attests rapid computing performance with high precision and accuracy in predicting the phenotype of unknown samples based on the trained model.

In this paper, we examined the efficacy of using a modular, multi-layer deep neural network for predicting host phenotype in comparison to other available machine learning tools. We found that our implementation provided a more robust and better performing model in most datasets.

## 2. Datasets

In order to test the efficacy of a deep neural network for phenotypic classification of taxonomic profiles, we collected metagenomic datasets from several independent research groups. We used these datasets for the experiment because MegaD should be evaluated by applying real-world datasets in different fields of healthcare. The first dataset was the cirrhosis dataset consisting of 232 metagenomic samples with 114 patient samples and 118 healthy control samples [30]. All samples from this dataset were obtained from people of Han Chinese origin. The samples from this dataset were sequenced using whole genome sequencing, and then turned into taxonomic abundance profiles using Kraken 2 software. There were 3302 taxonomic levels identified. The purpose of this dataset was to examine the efficacy of our tool on a small real-world dataset. The second dataset contained samples from patients with type II diabetes (T2D) and healthy controls. There were a total of 440 participants hailing from two different regions. There were 53 T2D samples and 43 control samples taken from a cohort of 70 year old European women. The samples from 344 Chinese T2D patients and non-diabetic controls are also included [31]. The purpose of this dataset was to benchmark our tool on a more robust real-world clinical dataset. The samples of this dataset were sequenced using whole genome sequencing, and then turned into taxonomic abundance profiles using Kraken 2 software. There were 606 taxonomic levels identified. The third dataset contained samples from canine specimens classified as obese or lean. This dataset was obtained from the Purina group and contained samples from 3096 individuals. Sequencing data was obtained from 1536 obese dogs, and 1560 lean dogs. A taxonomic abundance profile was generated from this sequencing data using the Kraken2 software tool. There were a total of 10,920 taxonomic levels identified. The purpose of this dataset was to serve as our ideal real-world dataset containing a large number of samples with very high read depth. Each dataset size of healthy control and patient samples and their accession numbers are shown in Table 1. The processed dataset for MegaD can be also found under the MegaD project website (https://github.com/BioHPC/MegaD (accessed on 27 April 2022)).

## 3. Methods

**Figure 1:**
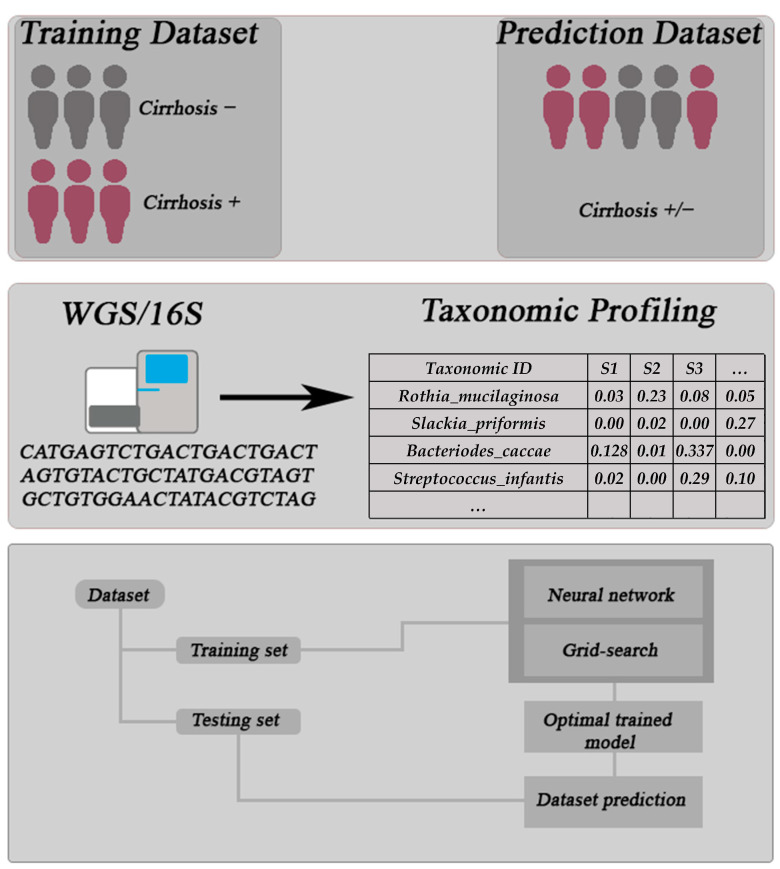
This figure shows the general workflow of our analysis and DNN tool from sampling to prediction. The first panel shows the selection of samples from a training and test dataset. The second panel shows the flow of sequencing of the samples to thereby produce a taxonomic profile. Finally, the last panel shows how the processed data is used in our tool to produce a predictive model for classification tasks.

Figure 1 shows a general overview of MegaD’s workflow. MegaD trains deep neural networks with taxonomic abundance profiles for classification of unknown sample profiles. All raw sequence data from samples in the datasets listed above are converted to taxonomic abundance profiles by utilizing the classification tools (1) QIIME2 for 16S data and (2) Kraken 2 or MetaPhlAn2 for WGS data. Given a sample, these classification tools identify bacterial species by comparing sequence reads with known organism genomes. All abundance profiles used are then converted into OTU table format for analysis.

For each of the datasets, metadata files are created by taking the sample IDs and disease status. The classifier takes as input a taxonomic table containing sample IDs and the metadata files created. The features extracted by the classifier are a two-dimensional vector for each sample ID containing the relative abundance of each of the taxonomic levels. The inputs are then split into training, validation, and testing datasets by using stratified splitting methods from the scikit-learn python package [32]. These datasets are then used to create a model for future classification of unknown taxonomic profiles.

**Figure 2:**
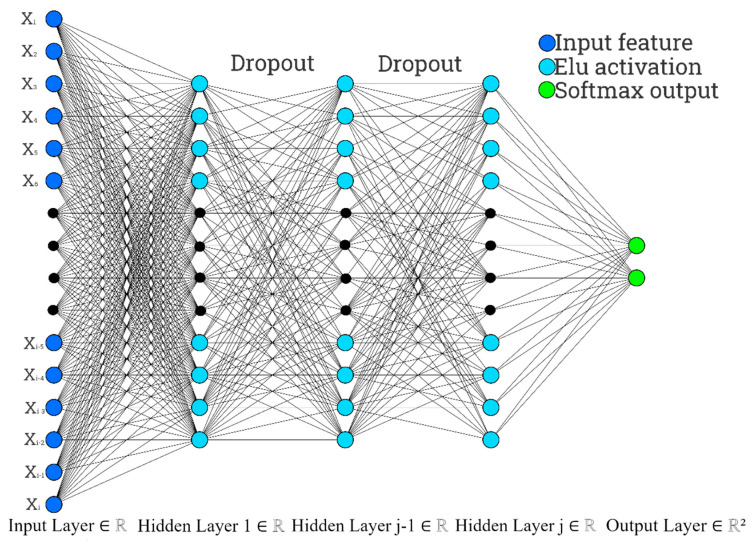
This figure shows a high-level representation of our deep neural network. The modularity of the network is represented by the black dots, which indicate a user-defined number of nodes in each layer. Additionally, although an arbitrary number of layers may be selected, only three were represented for this figure.

The machine learning model used in this project is a Deep Neural Network class created using the Pytorch and scikit-learn python packages. Deep Neural Networks utilize a series of connected layers each containing a set of perceptrons and weights that learn patterns within the training data through back propagation and stochastic gradient descent [33]. The main advantage of DNNs is their ability to handle very large datasets containing thousands of features while providing a very high accuracy [34]. The model uses a user-defined number of (1) neurons per layer and (2) hidden layers as shown in Figure 2. For each neuron, an activation threshold is calculated using an Elu activation function. Each linear layer is followed by a dropout layer which can be disabled based on user input. The final layer of the model is a softmax function to return class probabilities in a *M*x*N*array, where *M* is the batch size, and *N* is the number of classes. The model also employs the AdamOptimizer for gradient descent and a cross entropy loss function. We utilize a default learning rate of 0.00003 and a default weight decay of 0.0. We also implement batch gradient descent and a user-defined batch size with a default of 50. Finally, optional normalization of the data is performed using a cumulative sum scaling method. Once the DNN model is trained, the classifier computes the predictive accuracy with training, validation, and testing datasets and then outputs the results to the console. Additionally, precision, recall, and F1 score were calculated using the predicted vs. true labels for the test partition of each dataset.

Since many metagenomic datasets are vastly different from one another in terms of sample size and feature size, we have implemented a randomized grid search algorithm to simplify parameter optimization for the end user. This algorithm is designed to aim at the best results for each type of dataset, which could be either binary classification or multi-level classification. This randomized grid search runs through a user-defined number of iterations and, for each iteration, generates a random set of parameters from the predefined range. The parameters randomized by this method and the selected ranges are as follows: number of layers (5–75), number of neurons (5–75), dropout (True, False), dropout rate (0.1–0.9), learning rate (0.000005–0.0005), and weight decay (0–0.1). Additionally, the grid search method uses the validation accuracy to stop training appropriately when validation accuracy fails to improve over a user-defined number of random iterations, where its default value is configured to 10.

Finally, MegaD offers users the ability to predict an unknown dataset using a pre-trained model, or by using a novel training dataset. Once a model is selected or created, the user can load their unknown sample into the program, which will then return a class prediction based on the trained model. This feature is useful for identifying disease states of patients, providing a useful tool in precision medicine.

## 4. Results

In order to compare the efficacy of deep neural networks, we benchmarked our tool against the performance of three other analysis tools, such as MegaR, MetAML, and PopPhy. We measured the cross-validation accuracy of our model using the datasets Cirrhosis and type II diabetes (T2D), in order to compare against existing tools. For these datasets, we ran our tool using the grid search function to maximize accuracy of our model. The key selected parameters included a species level filter, with a 0.03 abundance threshold. In Table 2, our model performs a cross validation accuracy of 83.3% on the Cirrhosis datasets and 70% accuracy on the T2D datasets.

In MegaR and MetAML, the random forest algorithm was used for the comparison since this algorithm computed and created the best performing model of all. In PopPhy, the default convolutional neural network (CNN) was used for evaluation. We tested the performance of each tool on three datasets: (1) Cirrhosis, (2) type II diabetes, and (3) Purina obesity. The performance metric used to determine the efficacy of each program was prediction accuracy on a testing subset of the original dataset at a 15% stratified split. Accuracy was used since the dataset used is a balanced set.

Regarding the Cirrhosis dataset results, the highest accuracy 83.3% on the species taxonomic level was achieved by MegaD as shown in Table 2, where the integrated grid search function was used. We observed that this search function was performed as best parameters for each dataset. Regarding MegaR, the highest accuracy 88.5% on species taxonomic level was achieved by using the random forest model, where the parameters were set as follows: 0.05 threshold, 15% split, no normalization, and 1-201 variables. PopPhy and MetAML achieved 91% and 87.7% accuracy based on the default parameters, respectively. Then, we evaluated MegaD to determine the performance of its DNN classifier on the type II diabetes dataset. The performance of the DNN model of MegaD on this dataset was 70% accuracy, which is the highest accuracy compared to other analysis tools. MegaR, PopPhy, and MetAML achieved 67%, 65%, and 66.4% accuracy configured with the default settings, respectively. These results showed that our tool demonstrated a strong ability to predict disease status from taxonomic abundance profiles for either WGS or 16S sequencing data.

To determine the efficacy of our tool in disease status prediction from metagenomic samples, we tested our tool’s performance on the Purina dataset and compared the performance while configuring default parameters versus our grid search function. Our default parameters were: 10 layers, 25 neurons, an abundance threshold of 0.03, a learning rate of 1 × 10^−5^, normalization enabled, and taxonomic level set to species. The model was trained over 20 epochs. For our grid search results, all the settings were consistent at the default parameters with the exception of grid search set to true. In Table 3, MegaD showed 94.1% accuracy using the default parameters and 98.7% accuracy based on our grid search implementation. This experiment result signals a good indicator of our tool’s ability to predict the disease status of unknown samples using trained models.

**Figure 3:**
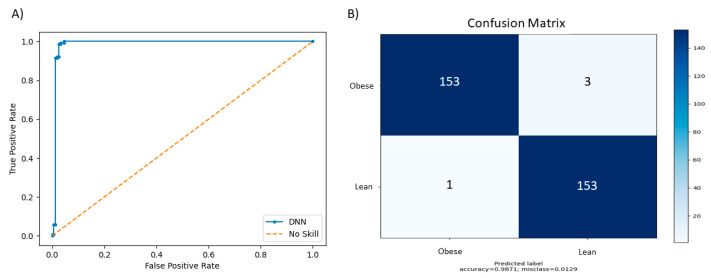
(**A**) This figure shows the ROC curve for our model on the Purina dataset using the grid search function. (**B**) This figure shows the confusion matrix which demonstrates the model’s misclassification rate for the Purina dataset broken down by true positive, false positive, false negative, and true negative.

In Figure 3, the performance of our DNN on the Purina dataset is shown by plotting the true positive rate against the true negative rate in an ROC curve graph. This graph demonstrates our model’s ability to make true positive predictions with high accuracy.

## 5. Discussion

Our results indicate that MegaD provides a quantitative improvement over other available tools on datasets with medium to large sample sizes, such as the Purina and T2D datasets. The main improvement of our tool is the ability to process very large datasets, such as the Purina dataset, which was unable to be run through the other tools tested. However, it does not seem to offer an improvement over other models on the Cirrhosis dataset, which we believe is due to the very small sample size of the dataset containing a total of 25 test samples. This is most likely due to the model not having enough training samples to properly generate an accurate model, while also not having enough testing samples to provide a stable accuracy metric. In comparison, the performance of MegaD on the Purina dataset shows that when given adequate training data, our tool is able to generate a highly accurate model capable of predicting unknown samples with strong confidence. It may be possible to improve our model’s ability to learn small datasets by providing an alternate option that uses a simplified network with reduced layers and nodes, which has been shown to perform better for smaller datasets by minimizing the amount of overfitting.

As a metagenomics analysis tool, MegaD provides notable improvements over currently available software tools. Most notably, MegaD offers comparable or better performance than other tools on the tested datasets which represent a wide range of different dataset types. Additionally, since MegaD offers the ability to save trained models and load them back in for rapid prediction, this allows for much faster and easier large-scale deployment of this tool in clinical settings. Although MegaD lacks the simplified user interface of tools, such as MegaR, it provides a simple enough interface and a robust set of instructions that allows use by those without a strong bioinformatics expertise.

Additionally, our results show that this method of phenotypic prediction may be useful as a diagnostic tool for certain ailments. With such a high accuracy in predicting obesity in dogs, we believe that there are other phenotypic predictions with distinct metagenomic profiles that can be diagnosed using our methods. Although we are unable to provide a reason for the metagenome of lean and obese dogs being unique, we are able to show that there is a correlation between the two that should be explored.

## 6. Conclusions

Utilizing a DNN classifier for phenotypic prediction of unlabeled taxonomic profile data showed very promising efficacy results. The main advantage of this type of classifier which we observed was the ability to function on very large datasets with many different levels of classification while maintaining a relatively high accuracy. This type of machine learning model also considerably reduces the wall-clock times of the prediction tasks compared to random forest and CNN models. In the benchmark experiments with three testing datasets, our deep neural network has performed better or similarly compared to the traditional models for phenotypic prediction. The implications of this work show that deep neural networks appear to be a more robust model for analyzing large and small metagenomic datasets. This paper shows strong evidence that DNN-based tools such as our tool allow users to rapidly perform large scale analysis of many different types of metagenomic data, while filling an important void in the field and opening the door to further research into the relationships between microbes and their environment.

## Figures and Tables

**Table 1:** Dataset breakdown for each of the three utilized datasets. Listed are the sample size and sample breakdown along with the accession number to access the raw data.

	Sample Size	Controls	Diseased	Accession #
Cirrhosis	232	118	114	ERP005860
T2D	440	217	223	SRA045646 SRA050230 ERP002469
Purina	3096	1536	1560	PRJEB20308

**Table 2:** Cross-validation accuracy for each software tool on Cirrhosis and type II diabetes datasets. Each tool was accessed on 27 April 2022.

Software	Software Available	T2D	Cirrhosis
MegaD (v1.0)	https://github.com/BioHPC/MegaD	0.700	0.833
MegaR (v1.0)	https://github.com/BioHPC/MegaR	0.670	0.885
PopPhy (v1.0)	https://github.com/YDaiLab/PopPhy-CNN	0.650	0.910
MetAML (v1.0)	http://segatalab.cibio.unitn.it/tools/metaml	0.664	0.877

**Table 3:** Performance of MegaD on Purina dataset using default parameters and grid search function. Listed are the accuracy, area under ROC curve, precision, and F1 score.

	Accuracy	AUC	Precision	F1 Score
Default Parameter	0.941	0.959	0.9103	0.940
Grid search	0.987	0.986	0.981	0.987

## Data Availability

https://github.com/BioHPC/MegaD/ (accessed on 27 April 2022).

## References

[1] Thomas T., Gilbert J., Meyer F. (2012). Metagenomics—A guide from sampling to data analysis. Microb. Inform. Exp..

[2] Huttenhower C., Gevers D., Knight R., Abubucker S., Badger J.H., Chinwalla A.T., Creasy H.H., Earl A.M., FitzGerald M.G., Fulton R.S. (2012). Structure, function and diversity of the healthy human microbiome. Nature.

[3] Qin J., Li R., Raes J., Arumugam M., Burgdorf K.S., Manichanh C., Nielsen T., Pons N., Levenez F., Yamada T. (2010). A human gut microbial gene catalogue established by metagenomic sequencing. Nature.

[4] Sanschagrin S., Yergeau E. (2014). Next-generation sequencing of 16S ribosomal RNA gene amplicons. J. Vis. Exp..

[5] Poretsky R., Rodriguez-R L.M., Luo C., Tsementzi D., Konstantinidis K.T. (2014). Strengths and Limitations of 16S rRNA Gene Amplicon Sequencing in Revealing Temporal Microbial Community Dynamics. PLoS ONE.

[6] Quince C., Walker A.W., Simpson J.T., Loman N.J., Segata N. (2017). Shotgun metagenomics, from sampling to analysis. Nat. Biotechnol..

[7] Jovel J., Patterson J., Wang W., Hotte N., O’Keefe S., Mitchel T., Perry T., Kao D., Mason A.L., Madsen K.L. (2016). Characterization of the Gut Microbiome Using 16S or Shotgun Metagenomics. Front. Microbiol..

[8] Afshinnekoo E., Chou C., Alexander N., Ahsanuddin S., Schuetz A.N., Mason C.E. (2017). Precision Metagenomics: Rapid Metagenomic Analyses for Infectious Disease Diagnostics and Public Health Surveillance. J. Biomol. Tech..

[9] Bolyen E., Rideout J.R., Dillon M.R., Bokulich N.A., Abnet C.C., Al-Ghalith G.A., Alexander H., Alm E.J., Arumugam M., Asnicar F. (2019). Reproducible, interactive, scalable and extensible microbiome data science using QIIME 2. Nat. Biotechnol..

[10] Liu B., Gibbons T., Ghodsi M., Treangen T., Pop M. (2011). Accurate and fast estimation of taxonomic profiles from metagenomic shotgun sequences. BMC Genom..

[11] Huson D.H., Auch A.F., Qi J., Schuster S.C. (2007). MEGAN analysis of metagenomic data. Genome Res..

[12] Ahn T.H., Chai J., Pan C. (2015). Sigma: Strain-level inference of genomes from metagenomic analysis for biosurveillance. Bioinformatics.

[13] Brady A., Salzberg S. (2011). PhymmBL expanded: Confidence scores, custom databases, parallelization and more. Nat. Methods.

[14] Patil K.R., Haider P., Pope P.B., Turnbaugh P.J., Morrison M., Scheffer T., McHardy A.C. (2011). Taxonomic metagenome sequence assignment with structured output models. Nat. Methods.

[15] Wood D.E., Lu J., Langmead B. (2019). Improved metagenomic analysis with Kraken 2. Genome Biol..

[16] Truong D.T., Franzosa E.A., Tickle T.L., Scholz M., Weingart G., Pasolli E., Tett A., Huttenhower C., Segata N. (2015). MetaPhlAn2 for enhanced metagenomic taxonomic profiling. Nat. Methods.

[17] Segata N., Waldron L., Ballarini A., Narasimhan V., Jousson O., Huttenhower C. (2012). Metagenomic microbial community profiling using unique clade-specific marker genes. Nat. Methods.

[18] Yassour M., Vatanen T., Siljander H., Hamalainen A.M., Harkonen T., Ryhanen S.J., Franzosa E.A., Vlamakis H., Huttenhower C., Gevers D. (2016). Natural history of the infant gut microbiome and impact of antibiotic treatment on bacterial strain diversity and stability. Sci. Transl. Med..

[19] Kostic A.D., Gevers D., Siljander H., Vatanen T., Hyotylainen T., Hamalainen A.M., Peet A., Tillmann V., Poho P., Mattila I. (2015). The dynamics of the human infant gut microbiome in development and in progression toward type 1 diabetes. Cell Host Microbe.

[20] Vatanen T., Kostic A.D., D’Hennezel E., Siljander H., Franzosa E.A., Yassour M., Kolde R., Vlamakis H., Arthur T.D., Hämäläinen A.-M. (2016). Variation in Microbiome LPS Immunogenicity Contributes to Autoimmunity in Humans. Cell.

[21] (2016). The MetaSUB International Consortium, The Metagenomics and Metadesign of the Subways and Urban Biomes (MetaSUB) International Consortium inaugural meeting report. Microbiome.

[22] Forbes J.D., Chen C.Y., Knox N.C., Marrie R.A., El-Gabalawy H., de Kievit T., Alfa M., Bernstein C.N., Van Domselaar G. (2018). A comparative study of the gut microbiota in immune-mediated inflammatory diseases-does a common dysbiosis exist?. Microbiome.

[23] Harris Z.N., Dhungel E., Mosior M., Ahn T.-H. (2019). Massive metagenomic data analysis using abundance-based machine learning. Biol. Direct.

[24] Pasolli E., Truong D.T., Malik F., Waldron L., Segata N. (2016). Machine Learning Meta-analysis of Large Metagenomic Datasets: Tools and Biological Insights. PLoS Comput. Biol..

[25] Luo D., Ziebell S., An L. (2017). An informative approach on differential abundance analysis for time-course metagenomic sequencing data. Bioinformatics.

[26] Dhungel E., Mreyoud Y., Gwak H.-J., Rajeh A., Rho M., Ahn T.-H. (2021). MegaR: An interactive R package for rapid sample classification and phenotype prediction using metagenome profiles and machine learning. BMC Bioinform..

[27] Reiman D., Metwally A.A., Sun J., Dai Y. (2020). PopPhy-CNN: A Phylogenetic Tree Embedded Architecture for Convolutional Neural Networks to Predict Host Phenotype from Metagenomic Data. IEEE J. Biomed. Health Inform..

[28] Oh M., Zhang L. (2020). DeepMicro: Deep representation learning for disease prediction based on microbiome data. Sci. Rep..

[29] Rodriguez-Brito B., Rohwer F., Edwards R.A. (2006). An application of statistics to comparative metagenomics. BMC Bioinform..

[30] Bajaj J.S., Betrapally N.S., Gillevet P.M. (2015). Decompensated cirrhosis and microbiome interpretation. Nature.

[31] Karlsson F.H., Tremaroli V., Nookaew I., Bergstrom G., Behre C.J., Fagerberg B., Nielsen J., Backhed F. (2013). Gut metagenome in European women with normal, impaired and diabetic glucose control. Nature.

[32] Fabian Pedregosa G.V., Gramfort A., Michel V., Thirion B., Grisel O., Blondel M., Prettenhofer P., Weiss R., Dubourg V., Vanderplas J. (2011). Scikit-learn: Machine Learning in Python. JMLR.

[33] Mishra P. (2019). Introduction to Neural Networks Using PyTorch.

[34] Mamoshina P., Vieira A., Putin E., Zhavoronkov A. (2016). Applications of Deep Learning in Biomedicine. Mol. Pharm..

